# Biomarkers and predictors for functional and anatomic outcomes for small gauge pars plana vitrectomy and peeling of the internal limiting membrane in naïve diabetic macular edema: The VITAL Study

**DOI:** 10.1371/journal.pone.0200365

**Published:** 2018-07-11

**Authors:** Matias Iglicki, Alejandro Lavaque, Malgorzata Ozimek, Hermino Pablo Negri, Mali Okada, Jay Chhablani, Catharina Busch, Anat Loewenstein, Dinah Zur

**Affiliations:** 1 Private Retina Service, University of Buenos Aires, Buenos Aires, Argentina; 2 NITIDO: Nuevo Instituto Tucumano de Investigación y Desarrollo en Oftalmología, Tucuman, Argentina; 3 Department of General Ophthalmology and Pediatric Ophthalmology Service, Medical University in Lublin, Lublin, Poland; 4 Eye Surgery Center Professor Zagorski, Lublin, Poland; 5 Diagnostic Ophthalmological Center, Buenos Aires, Argentina; 6 Royal Victorian Eye and Ear Hospital, Melbourne, Victoria, Australia; 7 Smt.Kanuri Santhamma Retina Vitreous Centre, L.V.Prasad Eye Institute Kallam Anji Reddy Campus, Hyderabad, India; 8 Department of Ophthalmology, University of Leipzig, Leipzig, Germany; 9 Division of Ophthalmology, Tel Aviv Sourasky Medical Center, Tel Aviv, Israel; 10 Sackler Faculty of Medicine, Tel Aviv University, Tel Aviv, Israel; 11 Incumbent, Sydney A. Fox chair in Ophthalmology, Tel Aviv University, Tel Aviv, Israel; Medizinische Universitat Graz, AUSTRIA

## Abstract

**Purpose:**

We aimed to investigate biomarkers and predictive factors for visual and anatomical outcome in patients with naïve diabetic macular edema (DME) who underwent small gauge pars plana vitrectomy (PPV) with internal limiting membrane (ILM) peeling as a first line treatment.

**Design:**

Multicenter, retrospective, interventional study.

**Participants:**

120 eyes from 120 patients with naïve DME treated with PPV and ILM peeling with a follow up of 24 months.

**Methods:**

Change in baseline best corrected visual acuity (BCVA) and central subfoveal thickness (CST) 1, 6, 12 and 24 months after surgery. Predictive value of baseline BCVA, CST, optical coherence tomography (OCT) features (presence of subretinal fluid (SRF) and photoreceptor damage), and time between DME diagnosis and surgery. Additional treatment for DME needed. Intra- and post-operative complications (cataract rate formation, increased intraocular pressure).

**Main outcome measures:**

The correlation between baseline characteristics and BCVA response (mean change from baseline; categorized improvement ≥5 or ≥10; Early Treatment Diabetic Retinopathy Study (ETDRS) letters) 12 and 24 months after surgery.

**Results:**

Mean BCVA was 0.66 ± 0.14 logMAR, 0.52 ± 0.21 logMAR, and 0.53 ± 0.21 logMAR (p<0.001) at baseline, 12 and 24 months, respectively. Shorter time from DME diagnosis until PPV (OR: 0.98, 95% CI: 0.97–0.99, p<0.001) was a predictor for good functional treatment response (area under the curve 0.828). For every day PPV is postponed, the patient’s chances to gain ≥5 letters at 24 months decrease by 1.8%.

Presence of SRF was identified as an anatomical predictor of a better visual outcome, (OR: 6.29, 95% CI: 1.16–34.08, p = 0.033). Safety profile was acceptable.

**Conclusions:**

Our results reveal a significant functional and anatomical improvement of DME 24 months after primary PPV, without the need for additional treatment. Early surgical intervention and presence of SRF predict good visual outcome. These biomarkers should be considered when treatment is chosen.

## Introduction

Diabetes mellitus is a major healthcare concern in people of working age. Worldwide, about 93 million are estimated to have diabetic retinopathy (DR).[1] Diabetic macular edema (DME) affects about 7% of diabetic patients and is the main cause for vision loss associated with DR.[2,3] In recent past, anti-vascular endothelial growth factor (VEGF) therapy has become first-line therapy for center-involved DME and are effective in improving and maintaining visual acuity, as shown in large-scale randomized controlled trials.[4–7]

In addition to the debilitating effect on their vision, DME patients suffer significant impairment of quality of life due to high treatment burden associated with intensive injection regimens. Over a 6-month period, DME patients have an average of 8.8 visits for their ocular condition—which come in addition to about 10 visits with other health care professionals.[8] DME treatment is associated with substantial direct medical costs for the patient, absenteeism for working patients and need for carer’s assistance for injection appointments.[8,9] Moreover, patients report anxiety and high expectations that lead to negative impact on long term anti-VEGF therapy and cause some delay in schedule a new appointment for intravitreal injection. Results from real-life studies are not comparable with the data known from randomized control trials, revealing that the actual number of anti-VEGF injections administered and the proportion of patients achieving significant BCVA gain are lower.[10,11]

The rationale to perform pars plana vitrectomy (PPV) in the treatment of DME is well explained by improvement of vitreous oxygenation in the context of ischemia due to diabetic retinopathy and hereby reduction of vitreous VEGF and cytokine levels.[12–14] Moreover, clearance of VEGF is increased after PPV.[15] Small gauge vitrectomy presents a proven treatment option in the current treatment of refractory DME.[16,17]

Furthermore, PPV effectively reduces macular thickness in cases of DME.[17,18]. The addition of ILM peeling to PPV has been shown to be beneficial for anatomical resolution of DME and VA improvement,[19–21] probably by avoiding the formation of secondary epiretinal membrane. Even in cases without apparent vitreomacular traction, complete release of the vitreoretinal interface and hereby inhibition of re-proliferation of fibrous astrocytes causes improvement of the condition in this advanced diabetic retinal disease. Although the addition of ILM peeling is controversial mainly due to a higher risk for complications,[22,23] the benefit outweighs the chances of intercurrences.[24]

When PPV is used as a ‘rescue’ procedure, treatment results are limited due to existing damage to the outer retina layers and the external limiting membrane.[21,25,26] The DRCR.net investigated the benefit of PPV in cases of DME with vitreomacular traction.[27]

Recently, PPV with ILM peeling has been described as a first line option in the treatment for patients with naïve DME.[18] However, in this study 20G vitrectomy was used and a relatively small number of patients with a short follow-up of 6 months was included. We are not aware of any study reporting the long-term outcome of primary PPV in DME and its predictors. Thus, we aimed to evaluate the role of small gauge PPV as a first line treatment for naïve DME with a follow-up of 24 months and to investigate biomarkers and predictive factors for visual and anatomical outcome.

## Methods

This was an international multicenter study involving 3 sites from (1) Private Retina Service, Buenos Aires, Argentina; (2) NITIDO Nuevo Instituto Tucumano de Investigación y Desarrollo en Oftalmología, Tucuman, Argentina; (3) Department of Ophthalmology, Medical University, Lublin, Poland (see S1 Text).

### Ethics statement

Institutional review board (IRB) approval was obtained through the individual IRBs at the participating institutes for a retrospective consecutive chart review. Approval for data collection and analysis was obtained from the IRB of the Buenos Aires ethics committee. The research adhered to the tenets of the Declaration of Helsinki. All data discussed in this study were fully anonymized before they were accessed. There was no need for informed consent.

Patient records from January 1, 2014 to December 1, 2016 were reviewed for cases of DME treated by PPV with ILM-Peeling as a first line treatment.

### Study participants

The following were set as study inclusion criteria: (1) age 18 years or older; (2) type 1 or 2 diabetes mellitus; (3) treatment-naïve DME causing visual loss (BCVA 20/40-20/200); macular edema defined clinically and by retinal thickness of >250 μm in the central subfield; and intra- or subretinal fluid (SRF) seen on SD-OCT; (4) treatment PPV and ILM-Peeling within 12 months from diagnosis of DME; (5) 24 months of follow-up after surgery.

Intravitreal therapy, as first-line therapy for DME were offered and discussed extensively with all patients.

Exclusion criteria were (1) other concomitant ocular disease that causes macular edema (i.e. neovascular age-related macular degeneration or choroidal neovascularization due to other reasons, retinal vein occlusion, uveitis and recent intraocular surgery possibly causing postsurgical macular edema); (2) any previous treatment for DME (i.e. anti-VEGF injections, intraocular corticosteroids, macular photocoagulation); (3) abnormalities of the vitreoretinal interface, such as epiretinal membrane, vitreomacular traction; (4) subfoveal atrophy or scarring as diagnosed clinically and on OCT.

Consecutive patient charts were reviewed for demographic data; HbA1c values; stage of retinopathy (diagnosed by clinical examination); best-corrected visual acuity (BCVA) and intraocular pressure (IOP) before surgery and after 6, 12, and 24 months; time between DME diagnosis and surgery (in days); use of IOP lowering treatment after 6, 12, 18 and 24 months; surgery details (PPV or combined cataract extraction with PPV); intra- and post-operative complications; any additional treatment after surgery; cataract progression after 12 and 24 months.

### Surgery procedure

25G PPV was performed using the CONSTELLATION Vision System (Alcon Laboratories, Inc.). In all eyes, a central vitrectomy was performed. The posterior vitreous was separated from the retina by active aspiration with the vitrectomy probe, and any visible vitreous strands that were adherent to the retina were removed. Intravitreous triamcinolone (40 mg/mL, Triesence^®^, Alcon, Forth Worth, Texas, USA) was systematically used in all cases as a marker to facilitate visualization and removal of the adherent posterior cortical vitreous. Triamcinolone was fully washed out before performance of ILM-peeling in all cases. ILM-peeling was systematically performed using vision blue G (0,125 mg Brilliant Blue G, Fluoron, Ulm, Germany) to stain and then remove the ILM. Postoperatively, topical antibiotic (Vigamox, moxifloxacin 0.5%, Alcon, USA) and antiinflammatory therapy (Pred Forte, prednisolone acetonide 1%, Allergan, Ireland) were administered 4 times daily over 1 month.

### OCT analysis

All included subjects were required to have OCT scans obtained using horizontal raster pattern scans centered on the fovea, obtained using spectral domain-OCT (Spectralis; Heidelberg Engineering, Heidelberg, Germany). Retinal thickness was analyzed using the retinal thickness map analysis protocol with nine Early Treatment Diabetic Retinopathy Study (ETDRS) subfields. Central foveal subfield thickness (CST) was defined as average retinal thickness of the circular area with 1 mm diameter around the foveal center and recorded at baseline and at 6, 12, and 24 months after surgery. Qualitative OCT analysis included the presence of subretinal fluid and damage of the photoreceptor layers at baseline and at 6, 12 and 24 months after surgery, graded by 2 masked assessors (MI and DZ).

### Outcome measures

Main outcome measures were the change of BCVA and CST at 12 and 24 months after the PPV and ILM-Peeling. Secondary outcomes were the proportion of eyes with ≥ 5 and ≥ 10 letters vision gain after 12 and 24 months, additional treatments needed, the proportion of cataract progression and extraction and intraocular pressure (IOP) lowering treatment during the study period. A subanalysis of patients that had a CST of <220μm (i.e. macular atrophy) after 24 months was performed.

### Statistical analysis

The demographics and clinical characteristics of our study cohort were evaluated using traditional descriptive methods. Changes in VA and CST from baseline were tested by paired t-test.

Univariate analysis for outcome measures (VA gain ≥ 10 letters, ≥ 5 letters) was done using t-test (for continuous variables) and Fisher’s Exact Test (for binary variables) by including the following variables: (1) age, (2) HbA1c, (3) duration of diabetes mellitus, (4) time to PPV, (5) BCVA, (6) CST, (7) MRT, (8) ISOS damage, (9) SRF and Lens status (phakic vs. pseudophakic) at baseline (10) and after 24 months (11). Predictors with a P value ≤ 0.001 in univariate analysis were included in the final Logistic regression model. A forward stepwise selection procedure was applied that retained only those variables with P < 0.05.

Statistical analysis was performed by the Statistical Laboratory School of Mathematics, Tel Aviv University, Tel Aviv, Israel. All statistics were computed with SPSS statistical package version 25.0.

## Results

The study included 120 eyes from 120 patients, with mean age of 67.0 ± 14.9 years. Demographic and baseline characteristics are detailed in Table 1.

**Table 1:** Patients characteristics.

Age, years, mean ± SD, (range)	67.0 ± 14.9 (25–99)
Male gender, n (%)	72 (60)
HbA1C, %, mean ± SD, (range)	8.8 ± 2.2 (5.9–16)
Diabetes mellitus duration, years, mean ± SD, (range)	16.6 ± 9.7 (2–56)
Time from DME diagnosis to PPV, days, mean ± SD, (range)	74.6 ± 76.5 (1–360)
s/p panretinal photocoagulation, n (%)	5 (4.2)
Pseudophakia, n (%)	60 (50)
BCVA, logMAR, mean ± SD, (range)	0.66 ± 0.14
Central subfield thickness, μm, mean ± SD, (range)	593 ± 92
Maximal retinal thickness, μm, mean ± SD, (range)	596 ± 84
Subretinal fluid, n (%)	79 (65.8)
IS-OS damage, n (%)	29 (24.2)

### Functional and anatomical outcome

The mean baseline BCVA was 0.66 ± 0.14 logMAR, improved to 0.52 ± 0.21 logMAR after 12 months (p<0.001) and remained stable over 24 months (0.53 ± 0.21 logMAR, p<0.001, Table 2). Fifty-seven (47.5%) and 52 patients (43.3%) gained ≥ 5 letters in vision after 12 and 24 months, respectively. Forty-three (35.8%) and 38 patients (31.7%) gained ≥ 10 letters in vision after 12 and 24 months, respectively.

**Table 2:** Functional and anatomical outcomes.

		P-value
BCVA baseline, logMAR, mean ± SD	0.66 ± 0.14	
BCVA 1 month, logMAR, mean ± SD	0.54 ± 0.18	<0.001
BCVA 6 month, logMAR, mean ± SD	0.52 ± 0.20	<0.001
BCVA 12 month, logMAR, mean ± SD	0.52 ± 0.21	<0.001
BCVA 24 month, logMAR, mean ± SD	0.53 ± 0.22	<0.001
CST baseline, μm, mean ± SD	600 ± 83	
CST 1 month, μm, mean ± SD	260 ± 33	<0.001
CST 6 month, μm, mean ± SD	240 ± 34	<0.001
CST 12 month, μm, mean ± SD	236 ± 31	<0.001
CST 24 month, μm, mean ± SD	230 ± 30	<0.001

BCVA—best corrected visual acuity; CST—central subfoveal thickness; SD—standard deviation; P-value—compared to baseline measurement.

Mean baseline CST was 593 ± 92 μm and decreased to 236 ± 31 μm at 12 months (p<0.001) and remained stable at 24 months (230 ± 30 μm, p<0.001, Table 2).

29 patients (24.2%) presented with damage to the IS-OS at baseline. Intact IS-OS layer at baseline correlated with improvement in BCVA after 24 months (p<0.001).

Forty-three patients (35.8%) presented with a CST < 220 μm after 24 months, as a sign of macula atrophy. A subgroup analysis revealed a significant worse final BCVA (0.60 ± 0.21 logMAR) in those eyes at 24 months compared to patients with final CST ≥ 220 μm (0.49 ± 0.22 logMAR, p = 0.015).

### Predictors for visual outcome

**Fig 1:**
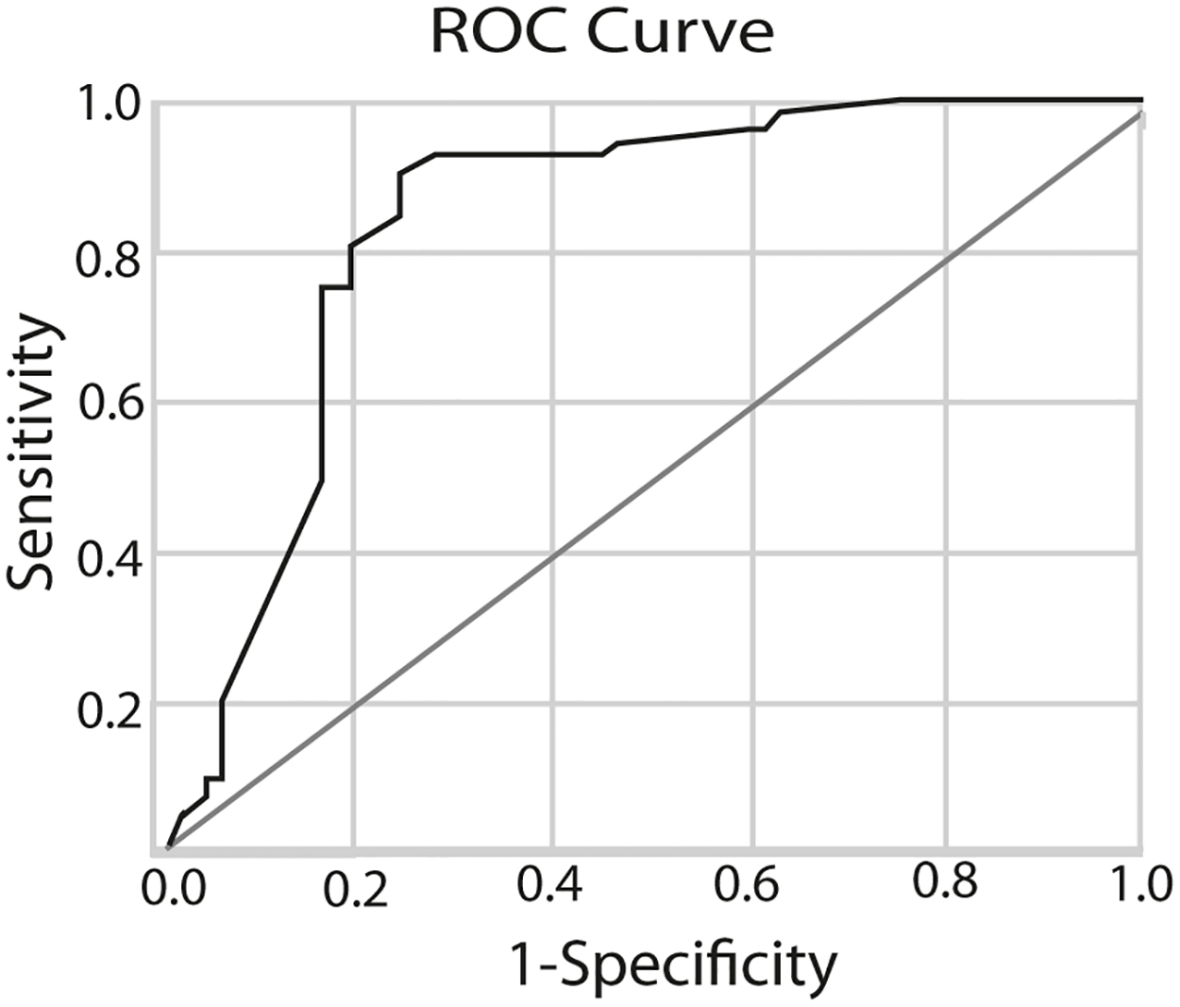
ROC curve for prediction of improvement in visual acuity following PPV with ILM-peeling. The sensitivity and specificity of timing until surgery and the presence of subretinal fluid in predicting the chance of gaining ≥5 letters BCVA 24 months after surgery. The area under the ROC curve was 0.828.

Shorter time from DME diagnosis until PPV (OR: 0.98, 95% CI: 0.97–0.99, p<0.001, Fig 1) was identified as clinical predictors for good functional treatment response (i.e. gain of ≥ 5 and ≥ 10 ETDRS letters, area under the curve 0.828). For every day PPV is postponed, the patient’s chances to gain ≥5 letters at 24 month decrease by 1.8%. Baseline HbA1C, duration of diabetes and lens status were not correlated with functional outcome (Table 3).

**Table 3:** Confounders for improvement in BCVA ≥ 5 letters after 12 and 24 months.

	12 months		24 months	
	Univariable Analysis P Value	Multivariable Analysis P Value	Univariable Analysis P Value	Multivariable Analysis P Value
Age	0.009		0.275	
HbA1C	0.253		0.668	
Diabetes mellitus duration	0.470		0.476	
Time to PPV	<0.001	<0.001	<0.001	<0.001
BCVA at baseline	0.256		0.427	
CST at baseline	0.505		0.179	
MRT at baseline	0.498		0.120	
ISOS damage at baseline	<0.001		<0.001	
SRF at baseline	<0.001		<0.001	0.033
Pseudophakia at baseline	0.093		0.093	

**Fig 2:**
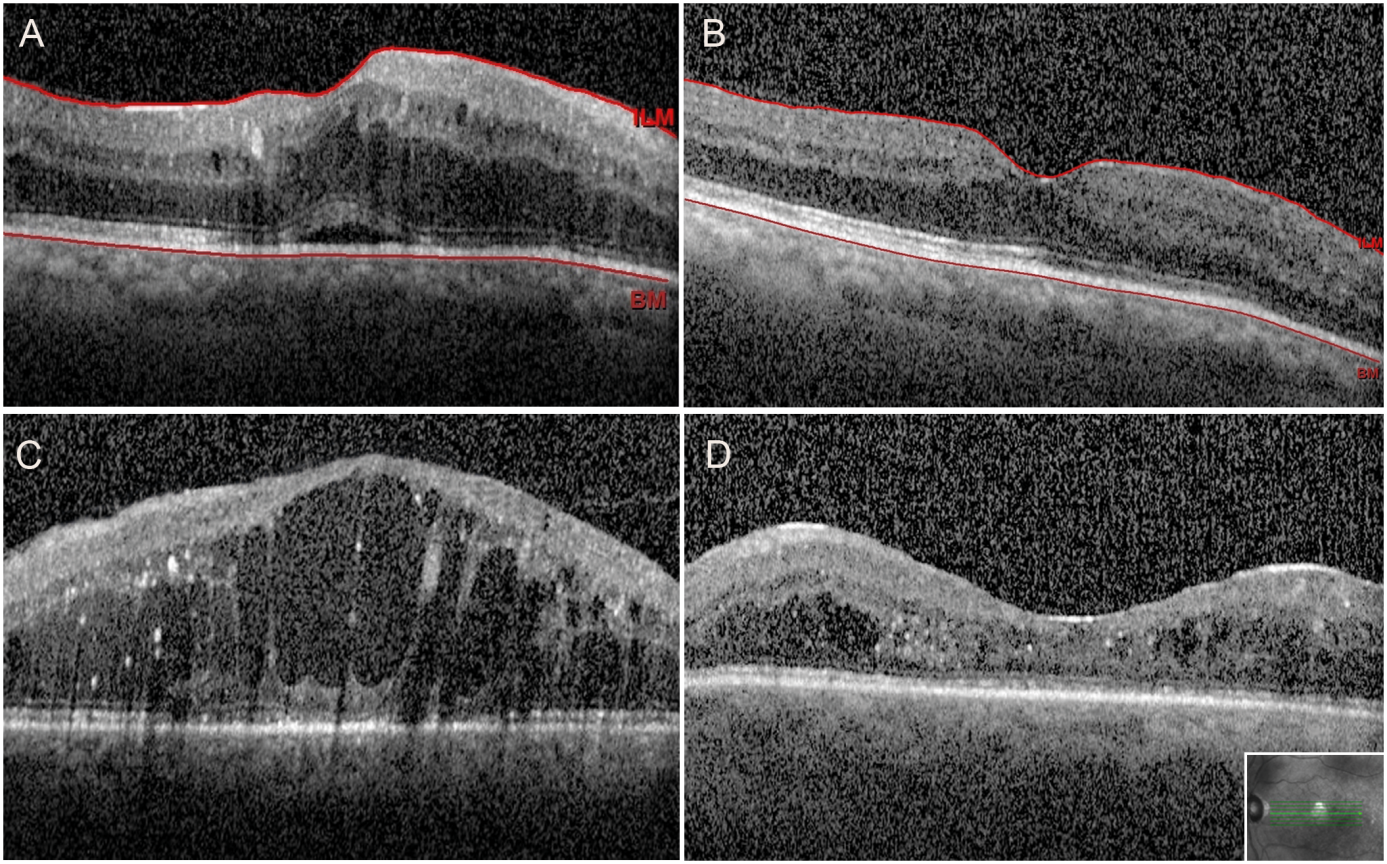
Biomarkers for visual outcome: OCT scans before and 24 months after PPV with ILM peeling. 2A-B. Patient presenting with biomarkers for good visual outcome, who had early intervention A. SD-OCT showing diffuse DME with subretinal fluid and intact inter-outer segment layer at baseline. B. 24 months after surgery complete resolution of intra- and subretinal fluid. 2C-D. Patient presenting without biomarkers for good visual outcome, who had late intervention. C. SD-OCT showing diffuse DME without subretinal fluid and damaged inter-outer segment layer at baseline. D. Persistent perifoveal intraretinal fluid and damaged inter-outer segment layer 24 months after surgery. Final CST measured <220 μm.

Presence of SRF was identified as an anatomical predictor of a better visual outcome, since patients with SRF at baseline were more likely to gain ≥ 5 letters after 24 months compared to those without SRF at baseline (OR: 6.29, 95% CI: 1.16–34.08, p = 0.033, Fig 2, Table 3). Baseline CST and MRT were not correlated with functional outcome.

In univariate analysis baseline CST and MRT were not predictive for functional outcome.

### Safety profile and additional treatments

Of 60 phakic patients (50%) at baseline, three patients (5%) underwent combined PPV with cataract extraction. All patients that remained phakic had cataract progression over 24 months. However, due to the fact that BCVA stayed stable they did not require cataract extraction. Lens status after 24 months showed no statistically significant influence on visual outcome (p = 0.344). None of the patients received any additional DME treatment including intravitreal therapy, macular and panretinal laser after PPV over the study period.

Twenty patients (16.7%) needed IOP lowering medication over 24 months. All were treated and well controlled with Timolol 0.5% twice a day. No patient needed surgical intervention for glaucoma.

Ten patients (8.3%) had intraoperative vitreous hemorrhage that resolved spontaneously within 15 days. One patient (0.9%) developed a full thickness macular hole, and another one a paramacular hole. Both were followed up without further intervention. Final BCVA at 24 months was 0.9 and 0.6 logMAR, respectively. Two patients (1.7%) developed postoperative rhegmatogenous retinal detachment and underwent repeated PPV with final BCVA of 0.9 logMAR.

## Discussion

We present a multicenter study of 120 eyes describing the functional and anatomical long-term outcome in patients with DME treated by primary PPV as first-line therapy. Our results reveal a significant functional and anatomical improvement 24 months after primary PPV, without the need for additional DME therapy (such as intravitreal treatment or macular laser) within the follow-up period. A previous report on primary PPV in DME patients only included a small number of patients treated with 20G PPV, which might be associated with an increased risk of intra- and postsurgical complications and only reported a 6-month follow-up.[18]

Forty-three percent and 31.7% of the patients gained ≥ 5 and ≥ 10 letters in vision, respectively. Results from real-life studies about intravitreal treatment for DME have shown an improvement of ≥ 5 and ≥ 10 letters in vision in 41–53% and 28–36% of cases treated by anti-VEGF therapy.[10,11] Hence, our data indicate a similar long-term outcome of primary PPV compared to intravitreal treatments in real-world conditions.

Timing of surgery was strongly correlated with functional results. For every day PPV is postponed, the patient’s chances to gain ≥5 letters at 24 month decreases by 1.8%. Those findings imply the importance of early therapeutic intervention in the treatment algorithm of DME. This phenomenon may be explained by the formation of outer retinal damage in long standing edema.[21] Indeed, in the current study, patients that had a longer time period between DME diagnosis and schedule for PPV procedure, presented more frequently with IS/OS.

The rationale of performing PPV in DME, even in this is the decrease of vitreous VEGF concentrations after PPV, causing a reduction in macular thickness in patients with DME.[12–14] Moreover, the diffusion of VEGF and other proinflammatory cytokines away from the macula is ameliorated after removal of the vitreous.[15] Accumulation of advanced glycation end-product in the diabetic vitreous leads to structural alterations of the posterior hyaloid and internal limiting membrane, strengthening the adhesion of the posterior vitreous cortex to the ILM.[28] Sustained hyperglycemia affects biochemical cascades, leading to destabilization of the vitreous gel.[28] Removal of the vitreous gel can have the additional benefit of decreasing the concentration of DME-promoting factors, such as AGEs, VEGF, and ICAM-1, which accumulate in the vitreous.[15,28] Some studies have also shown an improvement in fluid currents following vitrectomy, which may increase oxygenation of the inner retina.[29]

In this study, the presence of SRF was statistically significant as a predictor for functional outcome after 24 months and might prove as a biomarker.[30,31] In a previous report by our group, the presence of SRF and the absence of photoreceptor damage were identified as biomarkers for functional outcome of DME treated with dexamethasone implant.[31] The predictive value of SRF at baseline for treatment response to anti-VEGF agents in DME is discussed controversially. While some studies reported significant improvement in VA when SRF was present at baseline,[32,33] others found no difference or even an association with worse functional results.[34–36] In a post-hoc sub-analysis of the RISE and RIDE studies, the presence of submacular fluid predicted excellent visual outcomes in patients treated with ranibizumab.[32] However, in sham-treated patients, submacular fluid was associated with poor visual results and a four-fold risk of significant vision loss. These findings suggest that persistent serous macular detachment may have deleterious effects on visual function. As a clinical predictor a younger patients age was identified as a predictor for a good visual outcome, which is in concordance with previous report on functional outcome after anti-VEGF therapy.[32,37] It is speculated that photoreceptors in younger patients might better tolerate edema without incurring loss of visual potential. Prospective trials are needed in order to investigate the impact of age on functional outcome after PPV for naïve DME.

In the current study, 35.8% (43 eyes) ended with a CST < 220 μm after 24 months and significantly worse BCVA compared to eyes that remained a CST ≥ 220 μm. We hypothesize that this might be caused by the internal loss of homoestasis in DME due to Mueller cell damage. Similarly, Romano et al. reported macular atrophy after PPV with ILM-Peeling for DME, considering an intraretinal collapse of structural cells induced by ILM peeling.[22]

Twenty patients (16.7%) needed IOP lowering medication over 24 months. Delayed ocular hypertension has been reported previously after PPV.[38] Hyperoxygenation after PPV might play a role in the rise of IOP. PPV might increase exposure of the trabecular meshwork to oxygen levels.[39,40]

The major limitation of our study includes its retrospective nature and the lack of a control group. In the present study, diabetes duration was statistically correlated with photoreceptor damage at baseline. This is probably explained by prolonged glycemic exposure and longstanding unnoticed retinal disease before presentation. Importantly, HbA1C levels at any time point did not correlate with visual or anatomical outcome.

Over 24 months after PPV with ILM-peeling, none of the patients in this study needed any additional treatment, i.e. pharmacological therapy and, or macular laser photocoagulation. 43.3% and 31.7% of the patients gained ≥ 5 and ≥ 10 letters in vision, respectively. Even though a head-to-head trial comparing early PPV with ILM-peeling and anti-VEGF injections is needed in order to realize the role of surgery in the current treatment algorithm of DME, results from real-life studies about anti-VEGF treatment for DME have shown an improvement of ≥ 5 and ≥ 10 letters in vision in 41–53% and 28–36% of cases, respectively.[10,11] Therefore, in real-life conditions, visual outcome might be comparable using anti-VEGF therapy or PPV with ILM-peeling as a first-line option for the treatment of DME. Future randomized controlled clinical trials are needed in order to investigate the proper role of surgery in DME treatment.

## Supporting information

S1 Text (DOCX)

## Abbreviations

BCVA: best corrected visual acuity
CI: confidence interval
CST: central subfoveal thickness
DME: diabetic macular edema
ILM: internal limiting membrane
IRB: institutional review board
IS-OS: inner segment-outer segment
OCT: optical coherence tomography
OR: odds ratio
PPV: pars plana vitrectomy
SD: spectral domain
SRF: subretinal fluid
VEGF: vascular endothelial growth factor
VMT: vitreomacular traction
