# Supplementary material for: Biomarkers and predictors for functional and anatomic outcomes for small gauge pars plana vitrectomy and peeling of the internal limiting membrane in naïve diabetic macular edema: The VITAL Study
Source: PLoS One. 2018 Jul 11;13(7):e0200365. doi: 10.1371/journal.pone.0200365 (PMC6040739; doi:10.1371/journal.pone.0200365)
Supplement: S1 Text — (DOCX) [file pone.0200365.s001.docx]

Supplemental Material

**International Retina Group - Members**

- *Catharina Busch, MD*

Department of Ophthalmology, University of Leipzig, Germany

busch.catharina@gmail.com

### Jay Kumar Chhablani, MD

L.V.Prasad Eye Institute, Banjara Hills, HYDERABAD - 500 034 India

jay.chhablani@gmail.com

- Matias Iglicki, MD

University of Buenos Aires, Argentina

matiasiglicki@gmail.com

- Anat Loewenstein, MD

Division of Ophthalmology, Tel Aviv Sourasky Medical Center, Sackler Faculty of Medicine, Tel Aviv University, Tel Aviv, Israel

Incumbent, Sydney A. Fox chair in Ophthalmology, Tel Aviv University, Tel Aviv, Israel

[anatl@tlvmc.gov.il](mailto:anatl@tlvmc.gov.il)

- Mali Okada, MMed

Royal Victorian Eye and Ear Hospital, Melbourne, Victoria, Australia

okadam@gmail.com

- Dinah Zur, MD

Division of Ophthalmology, Tel Aviv Sourasky Medical Center, Sackler Faculty of Medicine, Tel Aviv University, Tel Aviv, Israel

[dinahzur@gmail.com](mailto:dinahzur@gmail.com)

**Participating Study Sites**

1. Retina Service; University of Buenos Aires, Buenos Aires, Argentina
2. Ophthalmology Division, Tel Aviv Sourasky Medical Center, Sackler Faculty of Medicine, Tel Aviv University, Tel Aviv, Israel
3. Department of Ophthalmology, Save Sight Institute, University of Sydney, Sydney, New South Wales, Australia
4. Department of Ophthalmology, University of Leipzig, Germany
5. NITIDO: Nuevo Instituto Tucumano de Investigación y Desarrollo en Oftalmología, Tucuman, Argentina
6. Department of General Ophthalmology and Pediatric Ophthalmology Service, Medical University in Lublin, Poland
7. Eye Surgery Center Professor Zagorski, Lublin, Poland
8. Diagnostic Ophthalmological Center, Buenos Aires, Argentina
9. Royal Victorian Eye and Ear Hospital, Melbourne, Victoria, Australia
10. Smt.Kanuri Santhamma Retina Vitreous Centre, L.V.Prasad Eye Institute Kallam Anji Reddy Campus, Hyderabad, India
